# Understanding Digital Mental Health Needs and Usage With an Artificial Intelligence–Led Mental Health App (Wysa) During the COVID-19 Pandemic: Retrospective Analysis

**DOI:** 10.2196/41913

**Published:** 2023-01-26

**Authors:** Chaitali Sinha, Saha Meheli, Madhura Kadaba

**Affiliations:** 1 Wysa Boston, MA United States; 2 Department of Clinical Psychology National Institute of Mental Health and Neurosciences Bengaluru India

**Keywords:** digital mental health, COVID-19, engagement, retention, perceived needs, pandemic waves, chatbot, conversational agent, mental health app, mobile health, digital health intervention

## Abstract

**Background:**

There has been a surge in mental health concerns during the COVID-19 pandemic, which has prompted the increased use of digital platforms. However, there is little known about the mental health needs and behaviors of the global population during the pandemic. This study aims to fill this knowledge gap through the analysis of real-world data collected from users of a digital mental health app (Wysa) regarding their engagement patterns and behaviors, as shown by their usage of the service.

**Objective:**

This study aims to (1) examine the relationship between mental health distress, digital health uptake, and COVID-19 case numbers; (2) evaluate engagement patterns with the app during the study period; and (3) examine the efficacy of the app in improving mental health outcomes for its users during the pandemic.

**Methods:**

This study used a retrospective observational design. During the COVID-19 pandemic, the app’s installations and emotional utterances were measured from March 2020 to October 2021 for the United Kingdom, the United States of America, and India and were mapped against COVID-19 case numbers and their peaks. The engagement of the users from this period (N=4541) with the Wysa app was compared to that of equivalent samples of users from a pre–COVID-19 period (1000 iterations). The efficacy was assessed for users who completed pre-post assessments for symptoms of depression (n=2061) and anxiety (n=1995) on the Patient Health Questionnaire-9 (PHQ-9) and Generalized Anxiety Disorder-7 (GAD-7) test measures, respectively.

**Results:**

Our findings demonstrate a significant positive correlation between the increase in the number of installs of the Wysa mental health app and the peaks of COVID-19 case numbers in the United Kingdom (*P*=.02) and India (*P*<.001). Findings indicate that users (N=4541) during the COVID period had a significantly higher engagement than the samples from the pre-COVID period, with a medium to large effect size for 80% of these 1000 iterative samples, as observed on the Mann-Whitney test. The PHQ-9 and GAD-7 pre-post assessments indicated statistically significant improvement with a medium effect size (PHQ-9: *P*=.57; GAD-7: *P*=.56).

**Conclusions:**

This study demonstrates that emotional distress increased substantially during the pandemic, prompting the increased uptake of an artificial intelligence–led mental health app (Wysa), and also offers evidence that the Wysa app could support its users and its usage could result in a significant reduction in symptoms of anxiety and depression. This study also highlights the importance of contextualizing interventions and suggests that digital health interventions can provide large populations with scalable and evidence-based support for mental health care.

## Introduction

The COVID-19 pandemic has had a detrimental impact on mental health outcomes worldwide [1-5]. The measures that were routinely implemented to control the spread of SARS-CoV-2, such as enforced self-isolation, quarantine, and social distancing, have contributed to the overall mental health impact of the pandemic by restricting daily lives and routines [6]. This psychosocial impact has resulted in an increase in the rates of loneliness, depression, anxiety, sleep difficulties, and substance use disorders [5].

The pandemic has created an overwhelming global public health emergency, with an increase in the rate of clinical disorders, through the triggering of new symptoms in individuals or the exacerbation of symptoms of pre-existing mental illnesses [3]. Given the fast-spreading nature of the virus, the pandemic quickly overwhelmed public health systems across the world, including mental health care facilities [5], and it has highlighted the gaps in health care systems rigorously. The tremendous challenges faced by countries globally in addressing the lack of health care facilities and personnel, combined with the pre-existing difficulties in accessing adequate and effective mental health care [7,8], pointed to an urgent need to look at additional support mechanisms.

Digitally delivered and self-guided mental health management interventions have emerged as an important response mechanism to this need [9,10]. These interventions have managed to provide more accessible mental health services that were otherwise disrupted by the pandemic, such as in-person therapy, and have also overcome pandemic-induced barriers to mobility and transportation. The pandemic thus became a turning point for digital mental health, with the world witnessing a surge in the usage of digital mental health interventions [11,12].

The increased usage of digital mental health interventions during the pandemic underlines the importance of further research on understanding the needs that prompted users to seek digital interventions; whether there were specific interventions that people were seeking; whether these interventions were efficacious; and whether any changes were evident in users’ needs, preferences, behaviors, and responses to digital mental health services due to the pandemic. This study aims to answer these questions through the analysis of real-world data from the users of Wysa—a digital mental health platform that leverages an artificial intelligence (AI)–based conversational agent as a means of deploying clinically safe and efficacious therapeutic support.

The objective of this study was to understand the utilization and efficacy of the Wysa app during the COVID-19 pandemic. To this end, this study aimed to (1) examine the relationship between mental health distress, digital health uptake, and COVID-19 cases; (2) evaluate engagement patterns with the app during this time period; and (3) understand the efficacy of the app in improving mental health outcomes for its users during the pandemic through self-reported pre-post assessments on the Patient Health Questionnaire-9 (PHQ-9 [13]) and Generalized Anxiety Disorder-7 (GAD-7 [14]) for symptoms of depression and anxiety, respectively.

## Methods

### App Background

Wysa is an AI-enabled, chat-based mental health app aimed at building resilience by fostering positive self-view and self-reflection. There are several components of the app that are built for this purpose, including a conversational agent and various intervention-based tools and techniques based on evidence-based practices, such as cognitive behavioral therapy, mindfulness, and guided behavioral reinforcement, and related approaches that have been found to be efficacious [15-18]. The app helps users tackle a variety of issues, including depression, anxiety, worry, loss, and other stressors. A study involving Wysa users reported a significantly higher average improvement in depressive symptoms among the high-engagement Wysa user group compared to that of the low-engagement Wysa user group [19]. Additionally, Wysa has been found to improve mental health outcomes in chronic conditions [20,21], with high engagement and adherence [22]. It was found that users were able to establish a strong therapeutic alliance with the AI-based conversational agent that was comparable to a human therapeutic bond [23], further confirming the positive feedback from users [24]. During the pandemic, Wysa also released 2 tool packs (or intervention packages) named “Health Anxiety” and “Remote Wellness,” which were contextualized to the pandemic. These were freely available to the public during the study period.

### Ethical Considerations

Wysa, which is publicly available on the Android and iOS app stores, was designed to prioritize safety, privacy, and security. The users downloaded the app after consenting to the app’s Terms of Service and Privacy Policy. Given that this study involved analyzing real-world data from an anonymous nonclinical population and was based on secondary deidentified data, it was exempt from registration in a public trial registry, according to the guidelines of the Office of Human Rights Protection [25]. No demographic information was collected from users, in accordance with data privacy and safety policies.

### Study Design

This study followed a retrospective observational design. Retrospective studies are not only used in health care studies but have also proven especially useful for COVID-19 studies, given the sudden onset of the pandemic [26-30]. The period of this study was set from March 1, 2020, to October 31, 2021, and users who joined on or between these two dates were included in this study.

### Measures

The number of installs; the number of emotional utterances of users; and usage data, such as the most used tools, number of tools started and completed, number of sessions, and number of days of engagement with the app, were examined to understand engagement patterns. The PHQ-9 and GAD-7 were used to measure the efficacy of Wysa in improving mental health outcomes for pre-post assessments. These questionnaires were administered within the app.

### Sampling Strategies and Inclusion Criteria

Users who installed the app during the study period were included in this study. The users included in the efficacy analysis (objective 3) were a subsample of the larger set of users included in this study. The users included in the subsample were those with moderate to severe symptoms on the PHQ-9 (n=2061) or GAD-7 (n=1995) and those who had completed their postassessment within 14 to 31 days of the first assessment. Users with a preassessment PHQ-9 score of ≥10 or a preassessment GAD-7 score of ≥8 were included.

### Analysis

#### Objective 1: To Examine the Relationship Between Mental Health Distress, Digital Health Uptake, and COVID-19 Case Numbers

The global mental health needs, in the situational context of the pandemic, were evaluated through the analysis of installation traffic data for a mental health app (Wysa) across 3 geographies and the emotional utterances from users who were situated in these geographies.

For each country, the pandemic wave durations and peaks were obtained from published government and scientific literature [31-33]. For the correlational analysis, a Pearson correlation coefficient was calculated, along with a *P* value at a 95% level of significance, for each country and their respective months in the study period via Python 3.6 (Python Software Foundation). The corresponding install numbers were mapped against the scaled number of active cases (divided by 100) to obtain a visual depiction for each country.

The emotional utterances during the waves of the pandemic were also examined for these countries. The proportion of distress-related utterances were examined and mapped in relation to the active cases of COVID-19 for visual depiction.

#### Objective 2: To Evaluate Engagement With a Mental Health App During the COVID-19 Pandemic

Engagement was assessed based on the number of sessions conducted during the study period and tool usage. The number of sessions conducted by users in the COVID-19 period were compared with the number of sessions conducted by randomized stratified samples of users from a prepandemic period (2018 and 2019). The sampling involved repeated replacement–based sampling, with 1000 iterations. A Mann-Whitney test was performed to evaluate the differences.

The engagement within Wysa was evaluated based on the frequency of starts and completions of individual tools (interventions) and tool packs (bundles of multiple interventions organized by need). Further, engagement with the tool packs released specifically for pandemic distress, which were titled “Remote Wellness” and “Health Anxiety,” was also evaluated.

#### Objective 3: Efficacy for Mental Health Outcomes

For the subsample of users who were included in the assessment of efficacy (objective 3), a Wilcoxon signed-rank test was performed to evaluate the differences among mean pre-post assessment scores on the PHQ-9 and GAD-7.

## Results

### Objective 1: To Examine the Relationship Between Mental Health Distress, Digital Health Uptake, and COVID-19 Case Numbers

**Figure 1:**
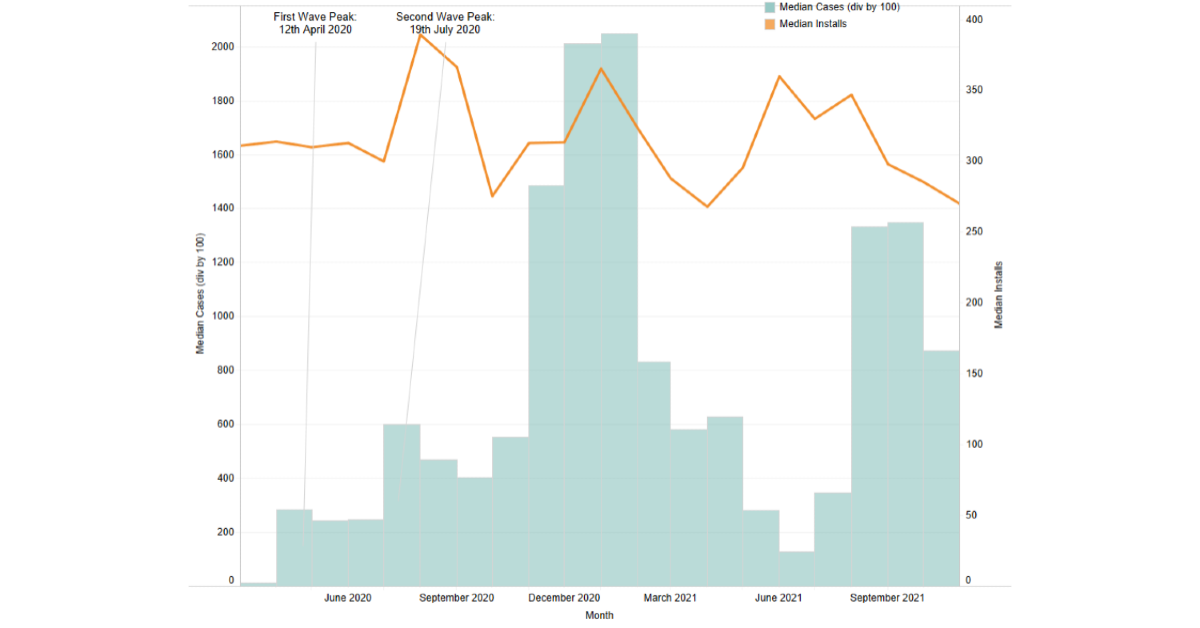
Installs corresponding to the pandemic wave peaks during the study period in the United States.

**Figure 2:**
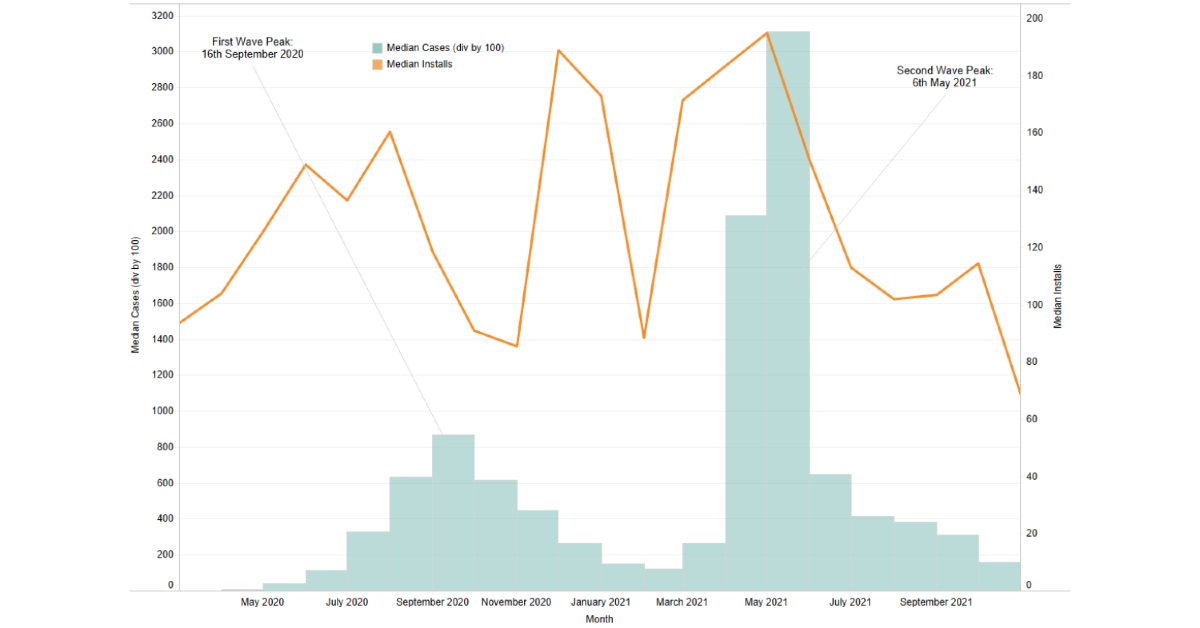
Installs corresponding to the pandemic wave peaks during the study period in India.

**Figure 3:**
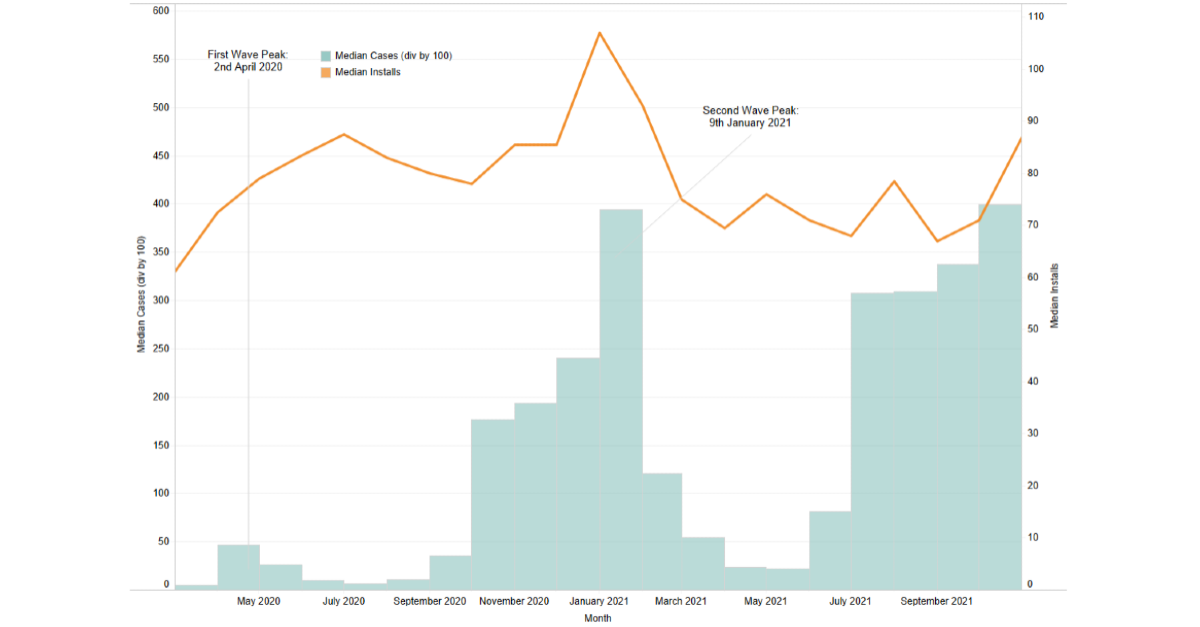
Installs corresponding to the pandemic wave peaks during the study period in the United Kingdom.

The assessed COVID-19 time period (March 2020 to November 2021) reflected a significant increase in the number of installations for Wysa globally. The rate of new installs, when mapped to pandemic wave peaks for specific geographies (United States, United Kingdom, and India), indicated that installs surged with the increasing case numbers (Figures 1-3).

When the relationship between COVID-19 active cases and new installs was evaluated, a significant positive correlation was observed in the geographies of the United Kingdom and India. For the United Kingdom, a positive correlation was observed for March 2020 (*r*=0.36; *P*=.049) and for April 2021 to June 2021, with June showing the strongest correlation (*r*=0.42; *P*=.02). Similarly, for India, a positive correlation was observed from March 2021 to August 2021, with August having the strongest correlation (*r*=0.61; *P*<.001). A weak negative correlation for the month of May 2021 (*r*=−0.43; *P*=.02) was observed for the United States, reflecting a delay between installs. The month-wise correlation values for each country are reported in Multimedia Appendix 1. For all 3 countries, an increase in installs corresponded with the peaks of COVID-19 waves, particularly during the second wave.

**Figure 4:**
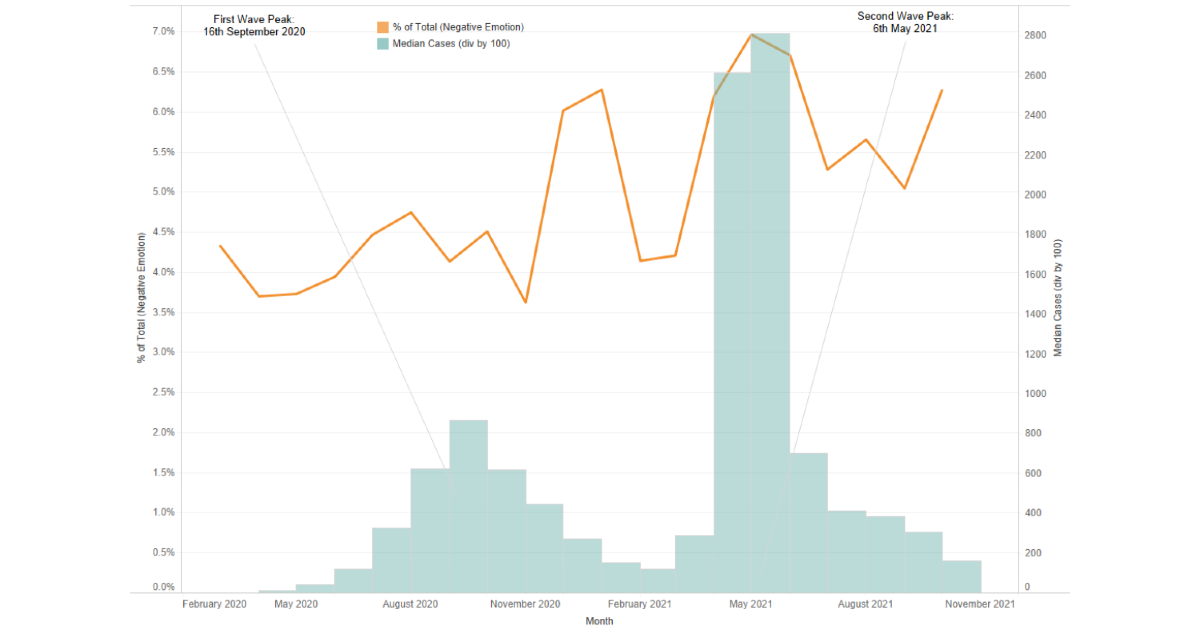
Proportion of distress-related emotional utterances corresponding to the pandemic wave peaks during the study period in India.

**Figure 5:**
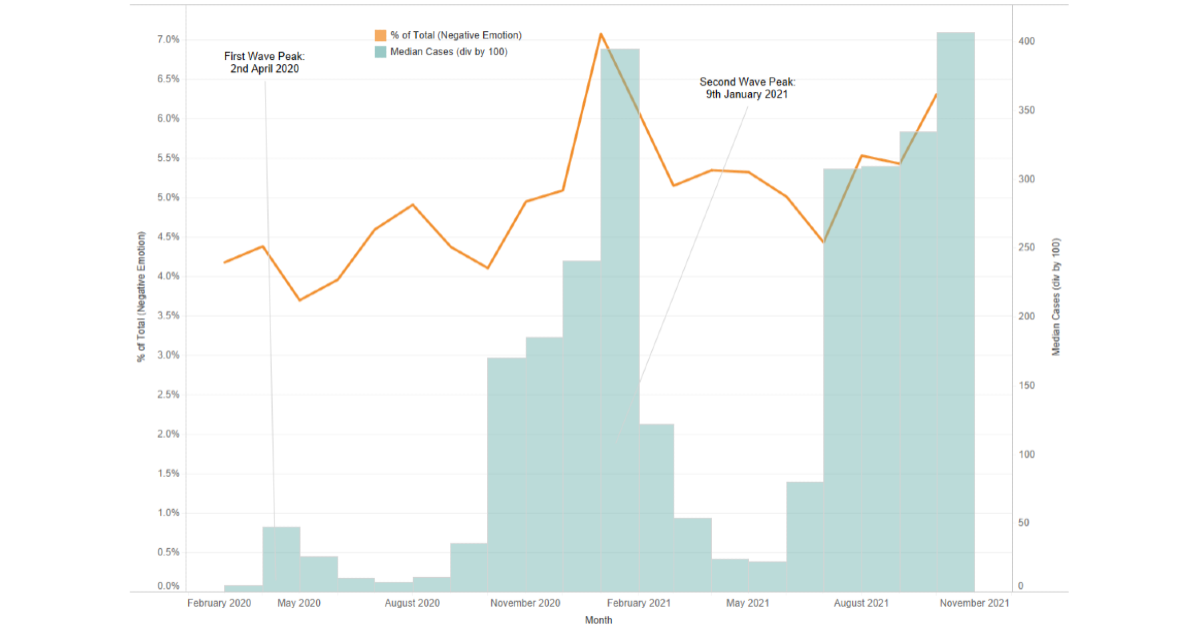
Proportion of distress-related emotional utterances corresponding to the pandemic wave peaks during the study period in the United Kingdom.

**Figure 6:**
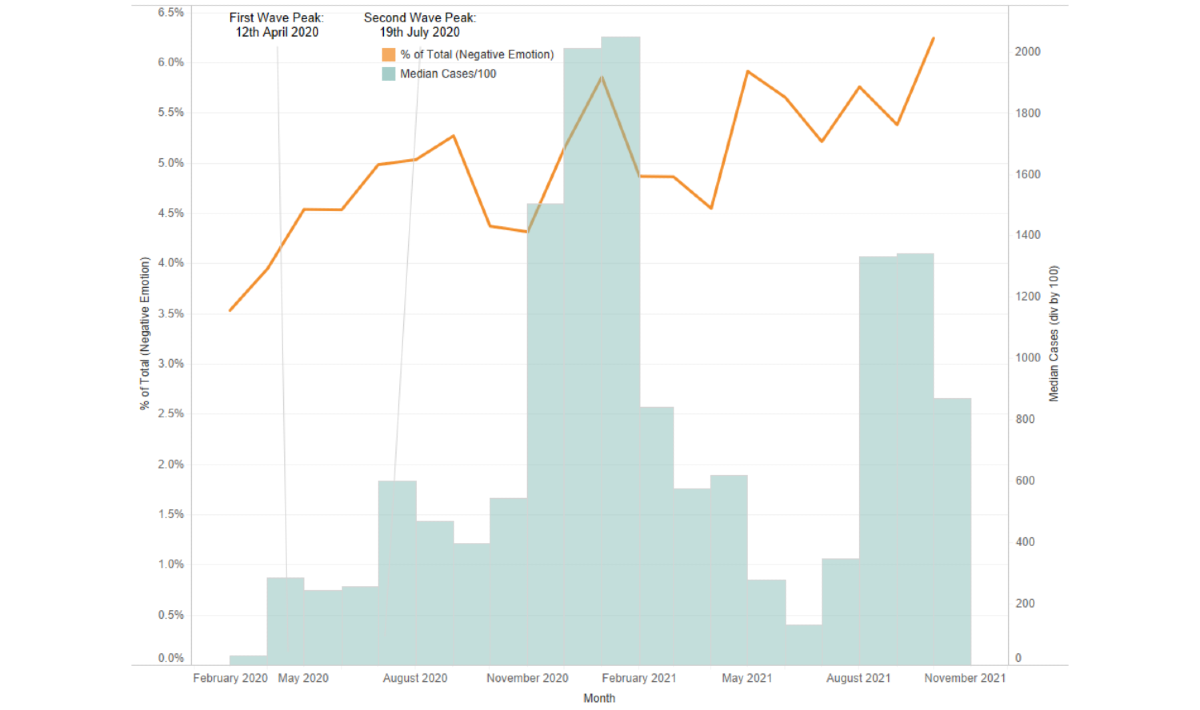
Proportion of distress-related emotional utterances corresponding to the pandemic wave peaks during the study period in the United States.

In an examination of the emotional utterances by users from the chosen geographies, the proportion of distress-related utterances (calculated based on the total number of utterances) corresponded with the crests of the COVID-19 case waves, and a continued high volume of distress-related utterances was present throughout the pandemic (Figures 4-6).

### Objective 2: To Evaluate Engagement With a Mental Health App During the COVID-19 Pandemic

**Figure 7:**
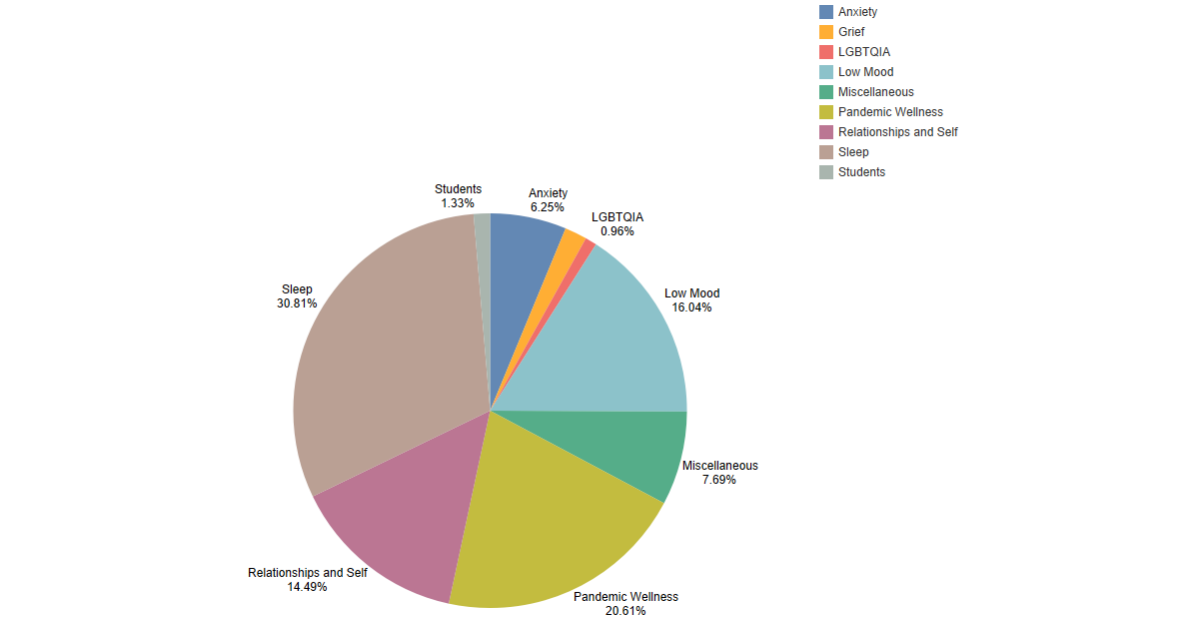
Engagement with different tool packs for users during study period (N=4541).

The intervention (tool) usage data of the COVID-period user sample (N=4541) indicated that the top five completed tools were those for managing anxiety, mindfulness, sleep stories, mindful compassion and sleep sounds. The most completed tool packs were those for managing sleep (30.81%, n=1400 ), pandemic wellness (20.61%, n= 936), managing low mood (16.04%, n=728) and interventions for relationships and self (14.49%, n=658) (Figure 7). Over a period of 15 days, this sample of users utilized an average of 29 conversations and interventions within Wysa. From the tool packs specifically released to manage pandemic-related concerns, the most used and completed tools were related to Self-Compassion (38.05%, n=374) and Managing Anxiety (28.76%, n=653).

The engagement (conversations with the conversation agent or chatbot) of these users was compared to that of 1000 iterative samples of pre–COVID-19 users. The COVID-19 period users displayed significantly higher usage and engagement with the app compared to those of the pre–COVID-19 users (Table 1), with a medium to large effect size for 80% of the iterations (all iterations can be found in Multimedia Appendix 2).

**Table 1:** Comparison of number of sessions (engagement) conducted by users from the COVID-19 period versus users from the non–COVID-19 period.

	COVID-19 period sample	Pre–COVID-19 period sample 1	Pre–COVID-19 period sample 2	Pre–COVID-19 period sample 3
Number of sessions, mean	18.85	12.3	11.74	12.11
Number of sessions, median	13.00	6.00	7.00	6.50
Mann-Whitney *U*	N/A^a^	349,653	340,984	344,394
*P* value	N/A	<.001	<.001	<.001
Effect size, *r*	N/A	0.6820 (medium)	0.6651 (medium)	0.6717 (medium)

^a^N/A: not applicable.

### Objective 3: Efficacy for Mental Health Outcomes

The PHQ-9 (n=2061) and GAD-7 (n=1995) analyses did not satisfy the criteria for normality. The Wilcoxon signed-rank test indicated significant differences in the pre-post assessments of the PHQ-9 (*P*<.001) and GAD-7 (*P*<.001) within the 95% CI, with medium to large effect sizes (Table 2).

**Table 2:** Wilcoxon signed-rank test for pre-post assessments of the Patient Health Questionnaire-9 (n=2061) and Generalized Anxiety Disorder-7 (n=1995).

	Depression	Anxiety
Wilcoxon signed-rank test, V	1,422,802	1,311,586
Bonferroni-adjusted *P* value	<.001	<.001
Effect size, *r*	0.569 (medium-large)	0.562 (medium-large)

## Discussion

### Principal Findings

This study aimed to use real-world data to observe the relationship between mental health needs, the COVID-19 pandemic, and the acceptability and efficacy of an AI-led mental health intervention (Wysa) for providing support.

The descriptive and correlational analysis found a significant relationship between an increase in mental health needs and the rise in COVID-19 active cases, as evidenced by the increase in new installs and distress-related utterances by users from the sample geographies. The number of installs increased for Wysa during the pandemic. This is consistent with recent literature, which suggests that there has been a rising number of installs for mental health apps during the pandemic [34].

The parallels between the increase in mental health needs and rising pandemic case numbers reflect the cascading impact of a greater volume of infections and its translation into psychological distress. These also indicate the multiple relationships between rising infections, behavioral responses, and public communication [35]. The rising number of distress-related utterances in each country correspond with observations made by other studies regarding the heightened presence of fear, uncertainty, and anger during the pandemic [36] and the observed increase of negative emotions on social media [37]. The relationship between case numbers and the number of installs and emotional utterances could also have been influenced by the effects of the policies and sociopolitical factors present in the studied countries (eg, the policies enacted to counteract the spread of the virus, including lockdowns, social isolation, and quarantines) [38,39]. Such invisible factors potentially had an impact on mental health needs that could explain the deviations and delays in the data, such as the sudden surges in the installation data that did not necessarily correspond to the case numbers of a given country (eg, those in the United States or the sudden rise of install numbers for India in late September 2020).

Research also suggests that with the advent of the pandemic, there was a growing feeling of comfort among users of digital support [40], which may be linked to the observed increase in installs throughout the course of the pandemic. It may also be speculated that while the onset of the pandemic played a crucial role in increasing the usage and adoption of technology [41], the consistent and continued use of technology is indicative of a larger shift in the acceptance of digital health [42].

The in-app engagement analysis indicated high usage during the COVID-19 period, with statistical significance observed in the intensity of engagement. Users heavily utilized health anxiety management interventions, especially in the context of contamination and illness fears [43]. Sleep concerns became more prevalent during the pandemic, as indicated by a comprehensive systematic review [44]. The engagement within this study reflected Wysa users’ needs and the ability of these users to seek support through the digital mental health intervention. The app’s most used tool packs included those that were contextualized for pandemic wellness, targeting the issues of stress, anxiety, low mood, and sleep. This study demonstrated their efficacy in improving mental health outcomes for symptoms of anxiety and depression. This evidence suggests that individuals can benefit from digital health interventions like Wysa and potentially find interventions more acceptable when they are tailored to specific contexts [45]. The demonstrated efficacy in improving symptoms of depression and anxiety further lends support to the important role that digital mental health apps like Wysa can play in effectively supporting individuals where there may be constraints on access or rising demand.

### Limitations

The findings of this study are unique but should be interpreted in the light of its limitations. For instance, this study is limited by the fact that we examined the installs only against the number of active cases and did not examine their alignment with other social or policy factors, such as closures or lockdowns. Further, the measurements for efficacy were made without a control group, and this could have increased the risk of regression to the mean [46]. The nonrandomized sampling of the retrospective design also limited the generalizability of the findings.

In the future, results regarding social indices and policy mapping can be further disseminated, and the broader scope of mental health needs can be understood through systematic studies that examine uptake and usage across the mobile health landscape.

### Conclusions

This study demonstrates the substantial increase in mental health uptake during the pandemic and its significant relationship with the rise in COVID-19 cases. It also demonstrates evidence for the efficacy of an AI-led digital health app in reducing symptoms of anxiety and depression for its users. This study sheds light on the importance of contextualizing interventions to the pandemic and lends further support to the important role that digital mental health apps can play in meeting the surging demand for mental health resources.

## Appendix

Multimedia Appendix 1 Month-wise correlation values for each country between pandemic waves and new installs.

Multimedia Appendix 2 All iterations of the engagement comparison between pandemic period and prepandemic period users.

## Abbreviations

AI: artificial intelligence
GAD-7: Generalized Anxiety Disorder-7
PHQ-9: Patient Health Questionnaire-9
